# The strike rate index: a new index for journal quality based on journal size and the h-index of citations

**DOI:** 10.1186/1742-5581-4-3

**Published:** 2007-04-19

**Authors:** William Barendse

**Affiliations:** 1 CSIRO Livestock Industries, Queensland Bioscience Precinct, 306 Carmody Road, St. Lucia, Queensland 4067, Australia

## Abstract

Quantifying the impact of scientific research is almost always controversial, and there is a need for a uniform method that can be applied across all fields. Increasingly, however, the quantification has been summed up in the impact factor of the journal in which the work is published, which is known to show differences between fields. Here the h-index, a way to summarize an individual's highly cited work, was calculated for journals over a twenty year time span and compared to the size of the journal in four fields, Agriculture, Condensed Matter Physics, Genetics and Heredity and Mathematical Physics. There is a linear log-log relationship between the h-index and the size of the journal: the larger the journal, the more likely it is to have a high h-index. The four fields cannot be separated from each other suggesting that this relationship applies to all fields. A strike rate index (SRI) based on the log relationship of the h-index and the size of the journal shows a similar distribution in the four fields, with similar thresholds for quality, allowing journals across diverse fields to be compared to each other. The SRI explains more than four times the variation in citation counts compared to the impact factor.

## Background

Measuring quality in science can be a difficult and lengthy process [1-7]. Verbal descriptions are probably best for describing the major achievements of individuals, with descriptors such as "revolutionised biology by formulating the theory of natural selection" to "honest toiler in the field". Although this is the favoured method of thinking of individuals, i.e., one does not introduce someone as Dr. Brown, whose work has been cited 3,000 times and who has an h-index [1] of 29 (or 29 articles cited 29 or more times), these qualitative statements of major achievements can be difficult to quantify. Further, choices often have to be made between individuals from different fields, or levels of remuneration have to be set, and some objective method is often required. While this ranking may be invidious [3], of journals, their scientific content and, by extension, the scientists themselves, it is also now quite firmly entrenched, and universal methods for assessing quality are required.

A first approach to a universal method of objective quantification of quality or usefulness would be to count citations, but using citation counts is not without its problems. Part of the difficulty in using citation counts is that different fields have different rates of citation. One has only to compare human genetics to cnidarian phylogeny (9940 vs 36 articles, highest citation count 1768 vs 112 see Methods). So comparing number of citations across diverse fields is inappropriate, although within a narrow field it may be the best way to quantify the impact of an article [2].

The impact factor of the journal suffers from the same limitation, that of differences in citation rate between fields, and although the data are normalised against the total number of citations that the journal receives, there are large differences in median impact factor between fields [8]. Many scientists and administrators use the impact factor to gauge scientific worth, or where to send their next manuscript [9], even if it is a poorer measure of quality of the individual article than the citation count [2,8], because impact factor is seen as a reliable guide to the performance of a journal within a particular field. Nevertheless, impact factor is a poorer measure of quality of the individual article compared to the citation count because most articles receive fewer citations per year than the impact factor of the journal they appear in [8,9], and there is usually a gap between a mean value and values contributing to that mean, so impact factor is always a crude replacement for the citation count of the individual article. Furthermore, a substantial amount of science can be published in several fields, and where the impact factor differs substantially between those fields, the impact factor by itself will not be a reliable indicator of the relative quality of two journals.

Although impact factor is a poorer predictor of quality of the individual article than citation count, impact factor, like any other index, has the advantage over citation count because one has to wait a few years for the overall citation count of an article to accumulate, and in the mean time it would be useful to know whether the journal has a reputation for quality. This is the advantage of an index, but indices should be comparable across fields. Although, the impact factor of the journal should not be used to compare journals in different fields [9], this need for comparability between fields has arisen through the setting of universal thresholds for acceptable impact factors for journals. This will clearly favour some fields, and may distort the editorial policies of some journals.

As one approach to a more universal or unbiased approach to ranking journals, I examined the h-index and journal size to derive a strike rate index for ranking journal quality. The h-index has recently been proposed [1,10] as an alternative method for the ranking of the output of a scientist, and is defined as the lowest rank of an article that has the same or more citations as its rank irrespective of what it is or where it is published. The characteristics of the h-index have been examined in depth [11,12] and it has been proposed as an index for journals [6,7]. However, journals vary greatly in size, so to apply it to journals one would need to take into account the very large differences that occur between publications, from quarterlies with irregular deadlines to weeklies with more than a dozen articles per issue. Publication sizes can range over 4 orders of magnitude; the h-index was formulated for scientists where productivity has a range of an order of magnitude and so differences in productivity may therefore be ignored in that context. Therefore a raw h-index could be expected to favour publications with a greater volume of articles and should be normalised in some way if it is to be used in a universal measure of journal quality. The characteristics of a strike rate index based on journal size and the h-index of the journal were examined, and it was applied to the citation counts in a narrow field of genetics to determine whether it was a better predictor of quality than impact factor.

## Methods

To calculate the h-index the articles to be compared are ranked in descending order of number of citations and *h *is the lowest rank for which an item has the same or more citations as its rank. An *h *= 100 means that there are 100 articles with 100 or more citations – if the 100^th ^ranked item has 105 citations but the 101^st ^has fewer than 101 citations then *h *= 100. All journals in Agriculture (AG), Condensed Matter Physics (CMP), Genetics and Heredity (GH) and Mathematical Physics (MP) were considered. These fields were chosen since they provide a four-way comparison, i.e., a field with low citations to that with high citations, and biological sciences compared to physical sciences. To obtain the h-index for a journal, the citation count for all its articles were obtained from ISI Web of Science in June 2006 for the period 1986 – 2006. The journal name was inserted into the General Search tool, which then returned all the articles for that journal. These were then sorted by number of citations and the h-index obtained by inspection. The search tool also gives the total number of items returned by the search, which is N. Journals sometimes change names, and all name changes were followed up and the new and old were combined together.

After analysis of the distribution of h vs N for these journals, the strike rate index (SRI) was formulated as 10log*h*/logN – multiplying by 10 gives an index between 0 and 10. The SRI need not be calculated on a 20 year time scale, it can be calculated for any arbitrary period, and rolling estimates may be a useful tool to evaluate changes in SRI; however, the shorter the period the more likely it is that the SRI will be biased towards articles of immediate attractiveness, and a shorter period would be expected to change any empirical thresholds for quality.

In this study, the citation count (CC) of articles in a narrow discipline are compared to the IF and SRI of the journals they appeared in. To make the comparisons accurate, CC for quantitative trait loci in *Bos taurus *(Cattle QTL), were examined. This was done to ensure that differences would be due to the qualities of the individual papers and the journals they occurred in rather than to any differences in popularity of the subject matter. Cattle QTL can be published in multidisciplinary (MD), GH and AG journals, which have very different impact factors (IF) [8].

The citations for Cattle QTL studies were counted in April 2006 using ISI Web of Science. Journals were classified as in the Journal Citation Reports into AG, GH or MD. For those journals that were listed under AG and GH, they were treated as GH. All citations were counted for articles published in 2003 and 2004, these years were chosen because that is what the 2005 impact factors would have been based on. Mean numbers of citations were compared using a one way analysis of variance with the P value obtained using 10000 permutations. Regressions were performed using the R statistical package [13]. Standard linear regressions of CC on IF or SRI were performed in a preliminary analysis. Due to the presence of at least one outlier – the outliers were different comparing SRI to CC and IF to CC – as determined using Cook's Distance [14] and the plot of the residuals against the quantiles of the standard normal, robust regressions were performed using the rlm tool of the MASS package in R [15] instead of removing the outliers. As CC, SRI and IF cannot have negative values, the intercept was constrained to be greater than or equal to zero.

To get a snapshot of citation in human genetics and cnidarian phylogeny, the two phrases were inserted into the TOPIC tool of the General Search of the ISI Web of Science on 16 March 2007. All articles were retrieved and then ranked by CC. The highest cited article that was actually about human genetics or addressed cnidarian phylogeny was determined. For human genetics this was the most cited article in the list, but for cnidarian phylogeny, this was the second most cited article, the first was about *C. elegans *molecular biology that happened to have the words cnidarian and phylogeny in the abstract.

## Results

**Figure 1:**
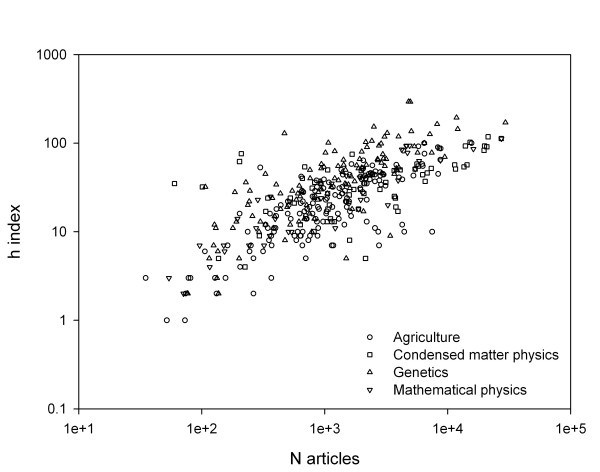
A double logarithm plot of the h-index and the number of citable items in a journal since 1986. The line of best fit has a slope of 0.57.

The h-index and N the total citable items were calculated for the 161 AG, 60 CMP, 124 GH, and 38 MP journals. The h-index and N show a linear relationship in a double logarithm plot (Figure 1), that is, the more a journal publishes the more likely it is to have highly cited works and a high h-index. The slope of this relationship was 0.57 and the R^2 ^was 55%. Journals from the four different fields cannot be separated into clusters in this plot. Journals with a high h-index for their size represent those with a higher than average track record for publishing articles that are well cited.

To illustrate the relationship between h-index, IF and N, the maximum h-indices for a GH and an AG journal were 295 and 100 respectively, with many AG journals having a higher h-index than GH journals – only 17 of the 124 GH journals had *h *≥ 100. Moreover, when an AG journal was matched to its nearest GH journal for both *h *and N, the GH journal had a higher impact factor. For example, the pair of the *Australian Journal of Agricultural Research *and *Animal Genetics *had almost identical *h *and N (44 & 2549 compared to 44 & 2429) but the IF were 0.993 and 2.437 respectively.

**Figure 2:**
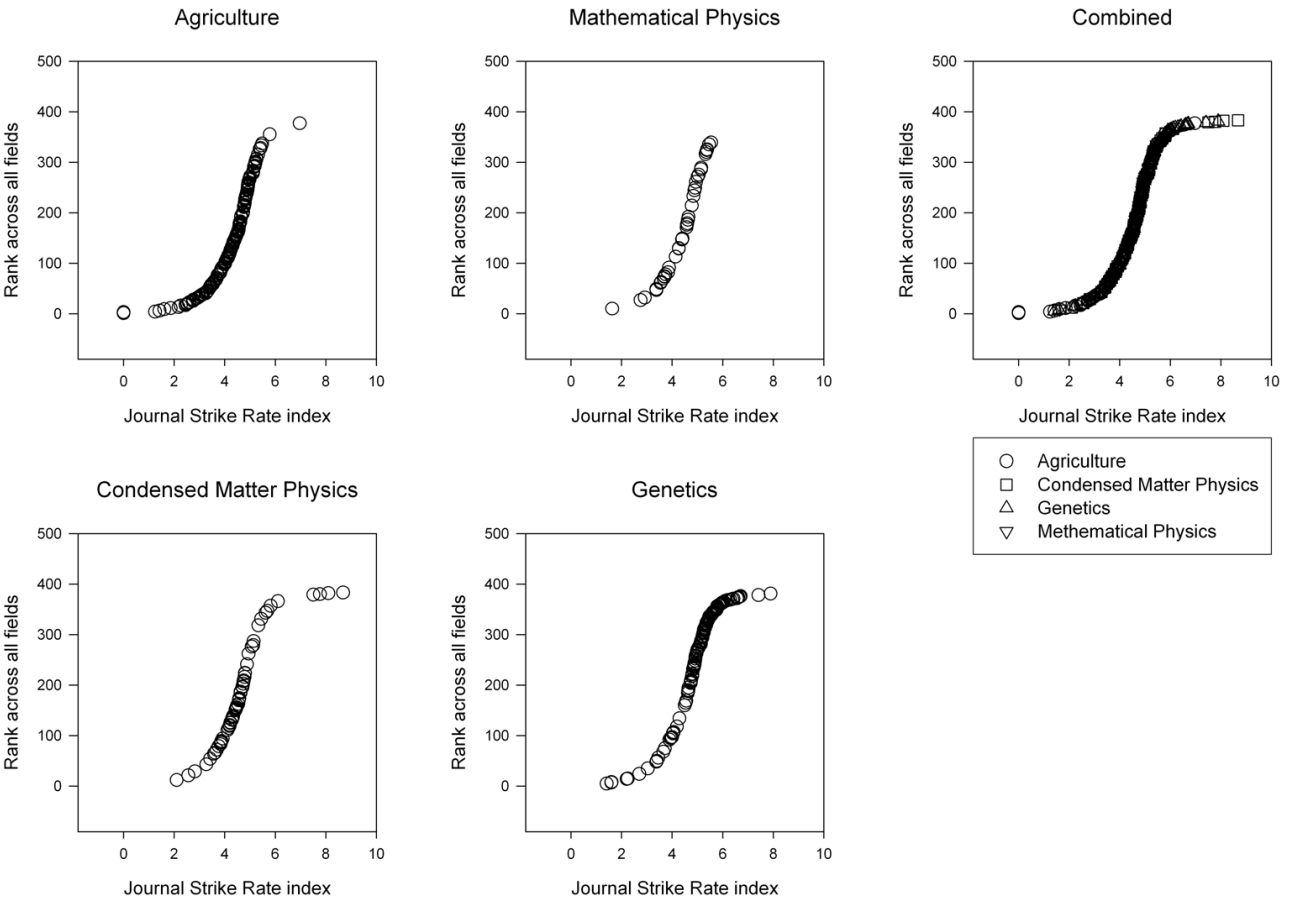
The ranking of the strike rate index across four fields.

To determine if there were any features of the SRI that might be common to all fields, the SRI for journals from all fields were ranked on one scale from 1 to 383 and plotted in ascending order (Figure 2). When all the journals are plotted on the one axis (Figure 2 combined) it is obvious that the graph has flat shoulders, or a bend at SRI ~ 4 and at SRI ~ 6, with most journals having an SRI between 3.0 and 7.0. Because this is a graph of ranks, it means that there are relatively few journals with SRI < 4 or SRI > 6. To determine whether the form of the graph was the same in all 4 fields, the SRI of each journal was separated into its field but was still plotted against its overall rank across all fields (Figures 2 Agriculture – Genetics). This allows the relative position of each journal to be seen clearly both within and across fields, as well as any consistent differences between fields. In all fields, most journals occurred between SRI ~ 4 and SRI ~ 6. A similar shape of the graph is seen in each field, although smaller fields show sparser plots. GH seems to have slightly more journals with SRI between 5 and 6, as can be seen in the density of the Genetics graph compared to the others. In contrast to the similarity between maximum and minimum SRI values in the different fields, the maximum IF in AG, CMP, GH and MP was 3.063, 17.857, 25.797 and 3.584 respectively.

The four fields showed similar median values for SRI but not similar medians for IF. Journals for AG, CMP, GH and MP showed medians of 4.4, 4.6, 5.1 and 4.5 respectively. This compares to median impact factors for these fields of 0.57, 0.97, 2.68 and 1.10 [8], a five fold range. There were more AG journals below 4, although the highest percentage was in MP (34%), and more GH journals above 6, although the two highest journals were review journals in CMP.

Most of the journals with SRI ≥ 6.0 were review journals (14 out of 20), most of which were in GH (n = 8). Most of the journals with SRI ≤ 4.0 were regional or published infrequently. Agriculture has, understandably, more regional journals than the other fields in both absolute numbers (41/161) and in percent, and has the largest number but not percent of journals with SRI below 4 – one should not generalise here since some of the best AG journals also have a country name in their title.

The SRI is not particularly biased against young journals in fast growing fields. *Genome Biology*, an Open, web based journal, is a case in point, 2005 is the first year it received an IF (9.712) and it has an SRI already of 5.34. While this is not yet up to that of *Genome Research *(IF 10.139, SRI 6.05), the journal is now in its 8^th ^volume. BMC *Genomics *is the highest of the other Open GH journals, with IF 4.092 and SRI 4.75, which compares to *Genomics *with IF 3.181 and SRI 5.45. While *BMC Genomics *has the higher impact factor it does not yet have the track record of *Genomics *of publishing highly cited articles.

**Figure 3:**
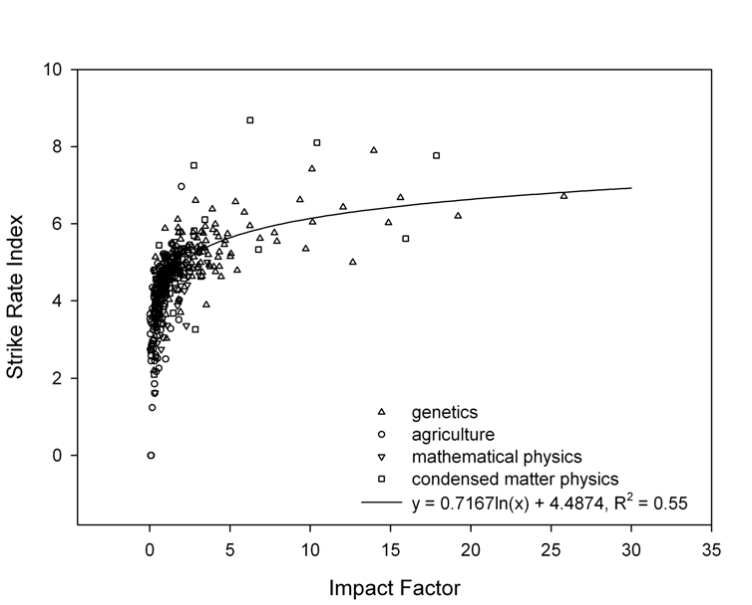
The relationship between strike rate index and impact factor across four fields.

Once the SRI is above approximately 4.5, the IF shows little relationship to the SRI (Figure 3) or the record of the journal for publishing highly cited work. Journals with very similar SRI will show markedly different IF. Journals in GH or CMP with very high IF did not show correspondingly higher SRI either to other journals in GH or CMP respectively, or to journals in AG or MP. Apart from those journals with high IF in these two fields, the plot of SRI by IF shows no differentiation between fields.

**Figure 4:**
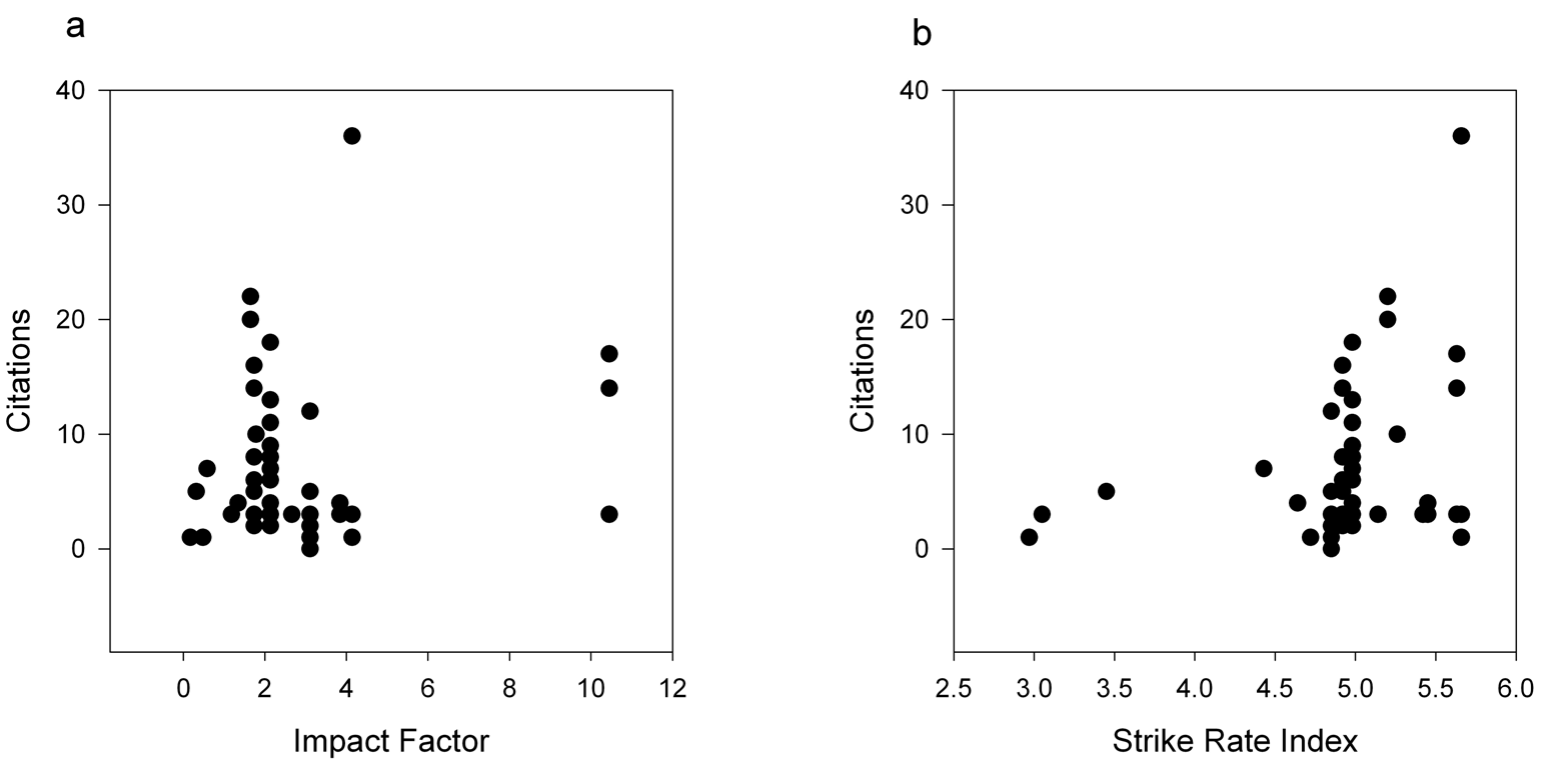
The relationship of citations for cattle QTL articles published in 2003–4 against (a) the impact factor and (b) the strike rate index of the journals in which they were published.

SRI explained a greater percentage of the variance in CC in cattle QTL studies than IF and, unlike IF, showed a significant slope of increasing CC with increasing SRI. There were a total of 375 citations to cattle QTL studies for 58 articles published in 2003 and 2004. The plot of CC versus IF is shown in Figure 4a. The slope of the robust regression of CC on IF was 0.34 (s.e. 0.30, t = 1.13 n.s.), and the model explained a non-significant 1.7% of the variance. Of these articles, 37 had between 0 and 5 citations and of these, eight had 0 or 1 citations. When grouped into journal type, the mean CC were significantly different. For citations for cattle QTL work published in 2003 and 2004, the mean CC for AG was 7.67 (N = 27, s.e.m. = 1.42), for GH was 4.79 (N = 28, s.e.m. = 0.99) and for MD was 11.33 (N = 3, s.e.m. = 4.26). These differences are significant with F = 2.3, P < 0.05. The CC for cattle QTL papers showed a strong trend when plotted against SRI (Figure 4b). The slope of the robust regression of CC on SRI was 1.09 (s.e. 0.12, t = 8.80 P < 0.001) and the model explained a significant 6.9% of the variance (P < 0.05). The SRI explained at least 4 times as much of the variation in CC compared to the nominal variation explained by IF.

## Discussion

The strike rate index showed some useful characteristics for a metric of journal quality that can be applied in any field of study. Firstly, the median values of the strike rate index of the fields tested in this study are very similar, while the median impact factors were 5 times larger in some comparisons. Secondly, extreme values were similar in the different fields, with most fields showing few journals below 4 or above 6; the highest impact factor in a field differs by 8 fold over the same fields, from IF = 3.063 to IF = 25.797. An SRI = 4.0 could be a lower limit for quality while that of SRI = 6.0 would correspond to extremely high quality or to review journals, the latter of which usually attract large numbers of citations. Indeed, it is of interest to see the strike rate index pick out review journals as a group. Nevertheless, it should be remembered that these thresholds are empirical and are based on a strike rate index calculated over 20 years. Shortening the number of years in the index would be expected to shift these thresholds. Thirdly, a linear double log relationship means that increases in the strike rate index should represent a linear quality scale.

The slope of the relationship between h-index and journal size shows that a journal should not increase its size just to increase the h-index, because the slope is less than 1. This means that the h-index does not increase as fast as the journal size, making it more difficult for extremely large journals to have a strike rate above 6, unless their editorial policies are highly selective. One example of a highly selective large journal is *Science*, with an SRI = 6.01. Journals may be better off improving quality first before increasing their size. Indeed, it appears that a journal should not be too young, too thin or hide itself away, but it should be fussy.

Not surprisingly, there were journals from Agriculture with low impact factor that contained articles with higher citation counts than journals from Genetics and Heredity with several times higher impact factor, although once the strike rate index was used, the citation counts were explainable, since these were well cited articles that appeared in highly ranked Agricultural journals such as the *Journal of Dairy Science *and the *Journal of Animal Science*. The strike rate index appeared to be more responsive to the rank or reputation of the journal in the field, which is often determined by the record of the journal in publishing highly cited articles.

The strike rate index and impact factor together provide a complementary analysis. High impact factors indicate journals that publish results that are highly relevant to a wide audience, at least in the short term, and may indicate those journals that do not publish work the editor thinks will be uncited in the first 2–3 years. The strike rate index identifies journals that maintain standards of content over the long term, irrespective of how those works may at first perform. A journal with a low impact factor but a high strike rate index would be one that put quality over immediate attractiveness, or was in a small field. One that had a high impact factor but an average strike rate index would be a journal that published work of immediate attractiveness but not long-term importance, or one that had an uneven or inconsistent policy of accepting manuscripts, or, perhaps, one that was a second tier journal in a very active field.

The strike rate index appears to identify journals that are superior in their field and to allow different fields to be compared without recourse to additional data. A good way to select journals is to rank them within a narrow field on impact factor, then ask how difficult is it to get published in that journal, how respected is the editor and their staff, who else publishes in that journal, and how long does it take to get published. All of that is valid, but once the impact factor is reified into a universal measure of journal ranking, those other aspects are apt to be forgotten. When organizations or governments set universal thresholds based on the impact factor, it can be hard for individual scientists to argue against them. The strike rate index helps to address the gap in knowledge of the meta-data associated with the publishing of science, by looking at the long term record of a journal in publishing highly cited material relative to the number of articles published.

## Abbreviations

AG Agriculture

CC Citation Counts or number of citations an article receives over its lifetime

CMP Condensed Matter Physics

GH Genetics and Heredity

h-index for items ranked in descending order of occurrence, it is the lowest rank of an item with the same or larger number of occurrences as its rank, so an *h *= 29 means the 29^th ^ranked article has 29 or more citations

IF impact factor, number of citations a journal receives for articles published in the previous two years, divided by a subset of the number of articles published in the previous two years; editorial and other brief notes are excluded from the denominator but not the numerator

MP Mathematical Physics

SRI strike rate index, measures the rate at which journals publish highly cited articles, calculated as 10log(h-index)/logN where N is all citable material in the journal

## Appendix

For a list of terms and explanations see Table 1

**Table 1:** 

Term	Explanation
Cook's Distance	measures the influence of a particular data point on all the other data points in a linear regression, it indicates how important a particular data point is for the method [14]
F	ratio of the variance or mean square between groups to the variance within groups
Linear Regression	where one variable is expressed as a function of another variable in a statistical analysis using simple least squares methods
log-log plot	double logarithm plot, if y = cx^a^, where x is the independent variable, c is a constant and a is an exponent, then logy = alogx + logc and the slope of the resulting line is the exponent a. An exponent of 2 would imply a square or quadratic relationship while an exponent of 0.5 would imply a square root relationship between the variables
median	half the values in a distribution are higher and half the values are lower than the median value
P value	with a null hypothesis of no difference between two or more samples, the P value is the probability that the null hypothesis is true, and that the observed difference is due to a chance event
Quantiles of the standard normal	QQplot. Plot of data against the corresponding quantiles of a standard normal distribution, one with a mean of zero and a variance of one. If the plot is fairly linear, the data are reasonably Gaussian or normal [16]
R^2^	the square of the correlation coefficient. It is an estimate of the variance explained by a particular statistical model
Robust Regression	the robust fit is minimally influenced by outliers in the data, minimizing bias in the estimates of the coefficients [15,16]
s.e.	standard error, which is an estimate of the accuracy of a mean (s.e.m.) or other coefficient given the variability found in a particular set of data; it is fundamental to understanding whether two means are likely to be from the same or from different distributions
t	calculated from the difference between means divided by the standard error of the difference between two means (Student's t-test) and in ordinary least-squares regression analyses to determine whether a slope is significantly different from zero by comparing the slope to its standard error
